# From searching for extraterrestrial biosignatures to exploring the origins of life: A prebiotic chemistry perspective

**DOI:** 10.1016/j.fmre.2026.01.017

**Published:** 2026-02-12

**Authors:** Fude Chen, Yufen Zhao, Jianxi Ying

**Affiliations:** a Institute of Drug Discovery Technology, Qian Xuesen Collaborative Research Center of Astrochemistry and Space Life Sciences, Ningbo University, Zhejiang 315211, China; b College of Chemistry and Chemical Engineering, Xiamen University, Fujian 361005, China

**Keywords:** Astrobiology, Origin of life, Prebiotic chemistry, Biosignatures, Chemical evolution

## Abstract

The search for extraterrestrial life represents one of humanity’s most profound scientific endeavors. This review examines the role of prebiotic chemistry in our quest to detect extraterrestrial life. Analyzing diverse chemical pathways, including the Strecker synthesis, cyanide polymerization, and Fischer–Tropsch-type reactions, outlines how simple inorganic precursors may evolve into complex chemical reaction networks under non-biological conditions. While compounds such as amino acids are common in both abiotic and biotic contexts, discerning their origins requires an understanding of reaction mechanisms, environmental contexts, and the thermodynamic challenges of polymerization. Moreover, this review addresses the emergence of homochirality and the development of genetic systems, proposing that these phenomena may arise from dynamic prebiotic processes. Laboratory simulations of extraterrestrial environments are crucial in testing these hypotheses, providing insights into the potential trajectories from simple monomers to complex macromolecules under planetary surface and interstellar conditions. Ultimately, this review advocates for reassessing biosignature interpretation methods, underscoring the need for integrated, multifaceted approaches that consider both prebiotic chemical evolution and environmental context when evaluating the potential for life beyond Earth.

## Graphical abstract

This review examines the role of prebiotic chemistry in our search for extraterrestrial life. Through the analysis of disparate chemical pathways for molecular biosignatures and laboratory simulations of extraterrestrial environments, we could provide insights into hypotheses concerning the chemical evolution of life under interstellar conditions.

**Figure:**
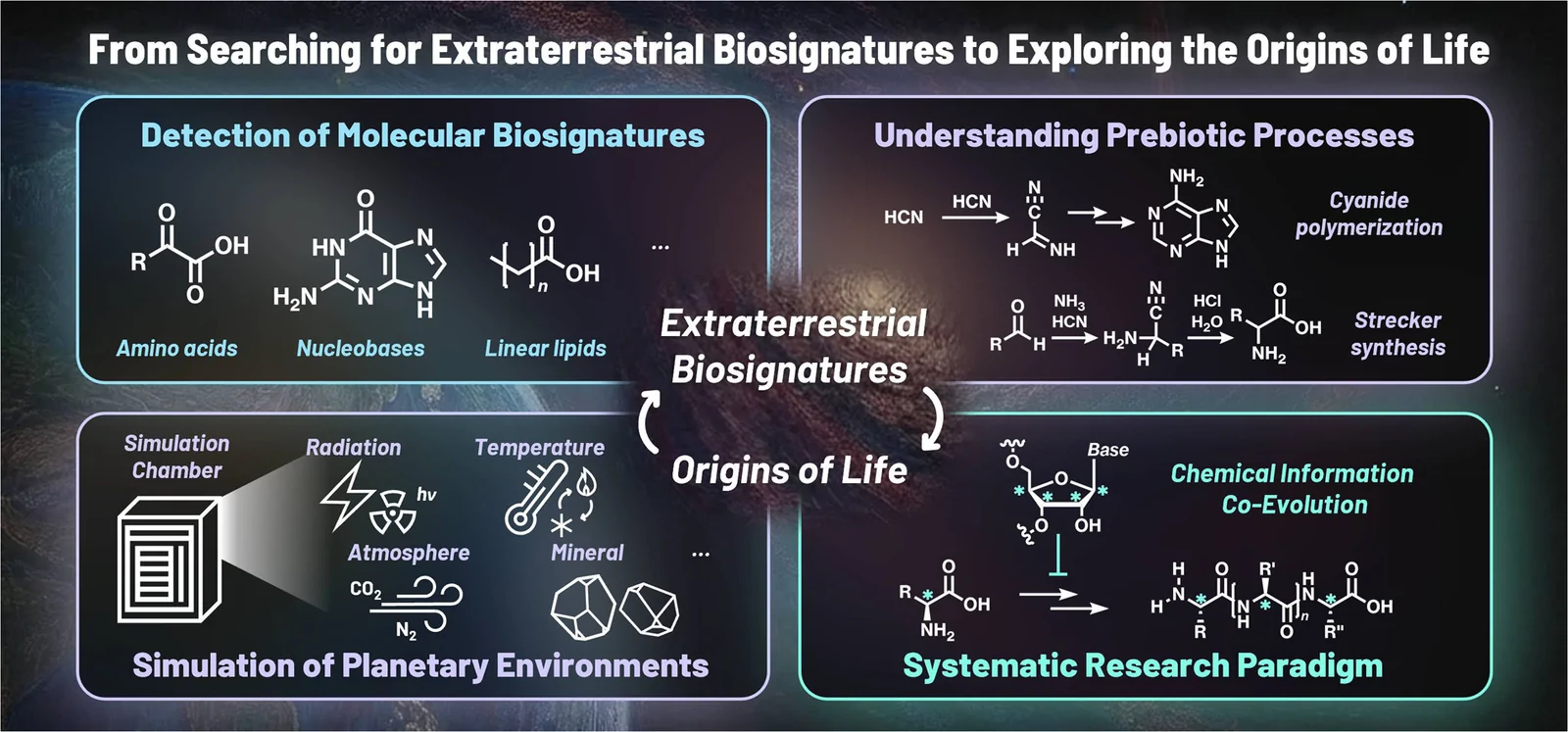

## 1 Introduction

The question of the origins of life is fundamental in both scientific and philosophical discourse. Understanding how life arose represents a critical component in determining whether a planet could have supported life in the past, maintained it in the present, or would harbor it in the future. With advancements in deep space exploration and sample-return missions, searching for signs of extraterrestrial life offers a direct way to address these questions. These searches extend beyond simple curiosity, providing critical insights into planetary habitability and the universal principles that govern the emergence of life.

Since the Viking missions of the 1970s [1], astrobiological research has progressively identified potential biosignatures on bodies including Earth, Mars, Venus, Enceladus, Ceres, and other exoplanets [[2], [3], [4], [5], [6]]. The most widely accepted definition of a biosignature is *any object, substance, or pattern whose origin necessitates a biological agent* [7]. Based on this definition, the range of potential biosignatures is extensive. Among the various classes of biosignatures, specific chemical compounds are considered particularly significant [8]. These molecular biosignatures, which range from simple hydrocarbons to complex structures such as amino acids and nucleotide precursors, can provide crucial evidence for prebiotic chemistry and may indicate the existence of extant or extinct life in extraterrestrial environments.

As with any single line of biosignature evidence, the presence of organic molecules does not, in itself, constitute definitive evidence of life [7,9]. The theory of chemical origin and evolution of life, as proposed by Oparin and Haldane, posits that life on Earth emerged through a gradual progression from simple chemical precursors to complex, evolving, and self-replicating systems [10,11]. Consequently, a central dilemma in astrobiology arises: must we only demonstrate the presence of these foundational molecules, or is it a prerequisite to show their integration into a complex chemical network capable of sustaining life? A comprehensive framework of prebiotic chemistry is therefore essential for evaluating the astrobiological implications of these findings.

Understanding the potential pathways by which life originates is fundamental to astrobiology, as it provides the essential context for interpreting potential biosignatures and guiding search strategies on other celestial bodies [7,12]. The field of prebiotic chemistry offers a crucial framework for this work. It investigates the synthesis of life’s fundamental building blocks, such as amino acids and nucleotides, from simpler inorganic molecules on a primitive Earth or other habitable worlds [13,14]. From the foundational molecules supplied by prebiotic chemistry, two principal paradigms propose pathways to cellular life: the “metabolism-first” and the “information-first”. The “metabolism-first” hypothesis posits the initial emergence of self-sustaining autocatalytic networks driven by geochemical energy, such as those theorized to exist in alkaline hydrothermal vents [15,16]. In contrast, the “information-first” paradigm argues that the formation of self-replicating informational polymers like RNA was the critical step, preceded by the abiotic accumulation of their constituent monomers [[17], [18], [19], [20]]. While both hypotheses offer compelling frameworks, the origin of life may not be a simple “either/or” process, but rather a synergistic evolutionary process of “metabolism” and “information” on a broader framework [21]. Thus, this review will focus on the molecular building blocks, surveying recent advances in the prebiotic synthesis of the fundamental molecular components of life.

Elucidating these specific prebiotic pathways is critical for astrobiological exploration. This knowledge enables scientists to evaluate whether an observed organic compound is a genuine biosignature or merely the result of nonbiological chemistry. To this end, laboratory experiments that simulate the diverse conditions of extraterrestrial environments are indispensable for testing these synthetic routes, thereby deepening our understanding of the origins of life and the possible conditions of the primitive Earth. Ultimately, this approach fosters a powerful feedback loop, where laboratory findings guide the search for extraterrestrial life, and new astronomical discoveries constrain models of prebiotic chemistry.

## 2 Biosignatures and plausible prebiotic synthesis pathways in extraterrestrial environments

The search for extraterrestrial life primarily focuses on detecting biosignatures that can be associated with the existence of life. Among all the chemical and physical biomarkers, molecular biosignatures that serve as building blocks for living organisms are particularly relevant to life as we know it. These compounds include amino acids, nucleobases, nucleotides, lipids, carbohydrates, and other metabolites (Fig. 1). Notably, extensive research demonstrates that many of these same compounds can be synthesized abiotically under conditions simulating early Earth or interstellar settings [20,22]. Consequently, their mere presence cannot be considered definitive evidence of life [7]. Despite this ambiguity, studying these abiotic pathways is vital, as they offer crucial insights into the prebiotic chemistry that sparked the origin of life.

**Fig. 1:**
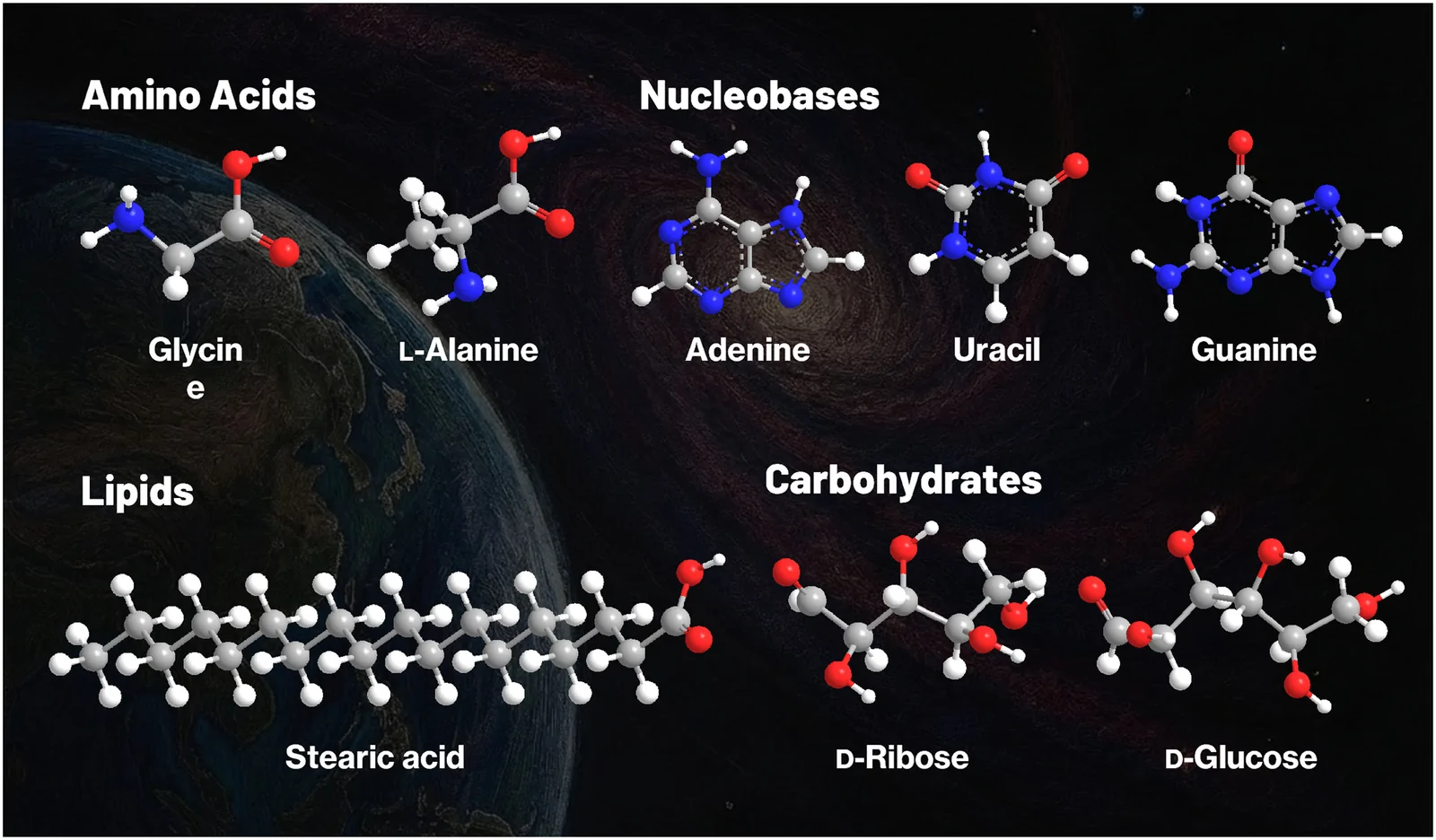
**Building blocks of modern life.** Several typical molecular biosignatures are building blocks for current life, including amino acids, nucleobases (nucleotides), lipids, and carbohydrates (sugars).

### 2.1 Amino acids and precursors of proteins

α-amino acids, characterized by a carboxylic group, an amine group, and a variable side chain attached to a central carbon, are critical to both metabolic functions and the structure of proteins. Their ability to condense into peptides by forming peptide bonds (or amide bonds) is a cornerstone in the evolution of complex biological machinery, particularly enzymes that drive metabolic processes. Although the role of proteinaceous amino acids is vital for living organisms, it is critical to recognize that non-protein amino acids may also undertake indispensable functions in both early metabolic networks and biochemistry.

Recent space missions and meteorite analyses have confirmed the presence of amino acids on a variety of extraterrestrial bodies [[4], [5], [6],^23^]. For instance, the Rosetta mission directly detected glycine, the simplest amino acid, within the coma of comet 67P/Churyumov-Gerasimenko [6]. Furthermore, carbon-rich chondrites, such as the Murchison meteorite [23], are particularly regarded as a rich repository of extraterrestrial amino acids. Analysis of samples from asteroid Ryugu has revealed the presence of over 20 different types of amino acids, which include both proteinogenic and non-proteinogenic varieties [5]. Similarly, the samples from asteroid Bennu, another carbonaceous asteroid, have been found to contain 33 amino acids, including 14 of the 20 proteinogenic ones [4]. Therefore, understanding the prebiotic pathways that led to the formation of these molecules is as critical as their detection and analysis.

Since the Miller-Urey experiment first produced amino acids and other organic compounds from inorganic reactants in 1953 [24], abiotic or prebiotic pathways for the formation and evolution of amino acids have been reported from various perspectives. The Strecker amino acid synthesis (Fig. 2a) has been considered the primary mechanism for the formation of α-amino acids in meteorites [25]. This process begins with the formation of an imine through the condensation of an aldehyde and ammonia, followed by the cyanide ion attacking the iminium carbon to form an aminonitrile. After the complete hydrolysis of the cyano group, an α-amino acid results [26]. Strecker synthesis can explain the formation of a broad range of amino acids (with different R-groups) from simple aldehyde precursors. However, it cannot directly produce other forms of amino acid, such as β-amino acids, while Michael addition reactions with α,β-unsaturated nitriles and ammonia can yield [27]. Such alternative processes will broaden our perspective on the potential diversity of prebiotic molecules and underscore that multiple concurrent mechanisms might have contributed to the observed chemical biosignatures in extraterrestrial samples.

**Fig. 2:**
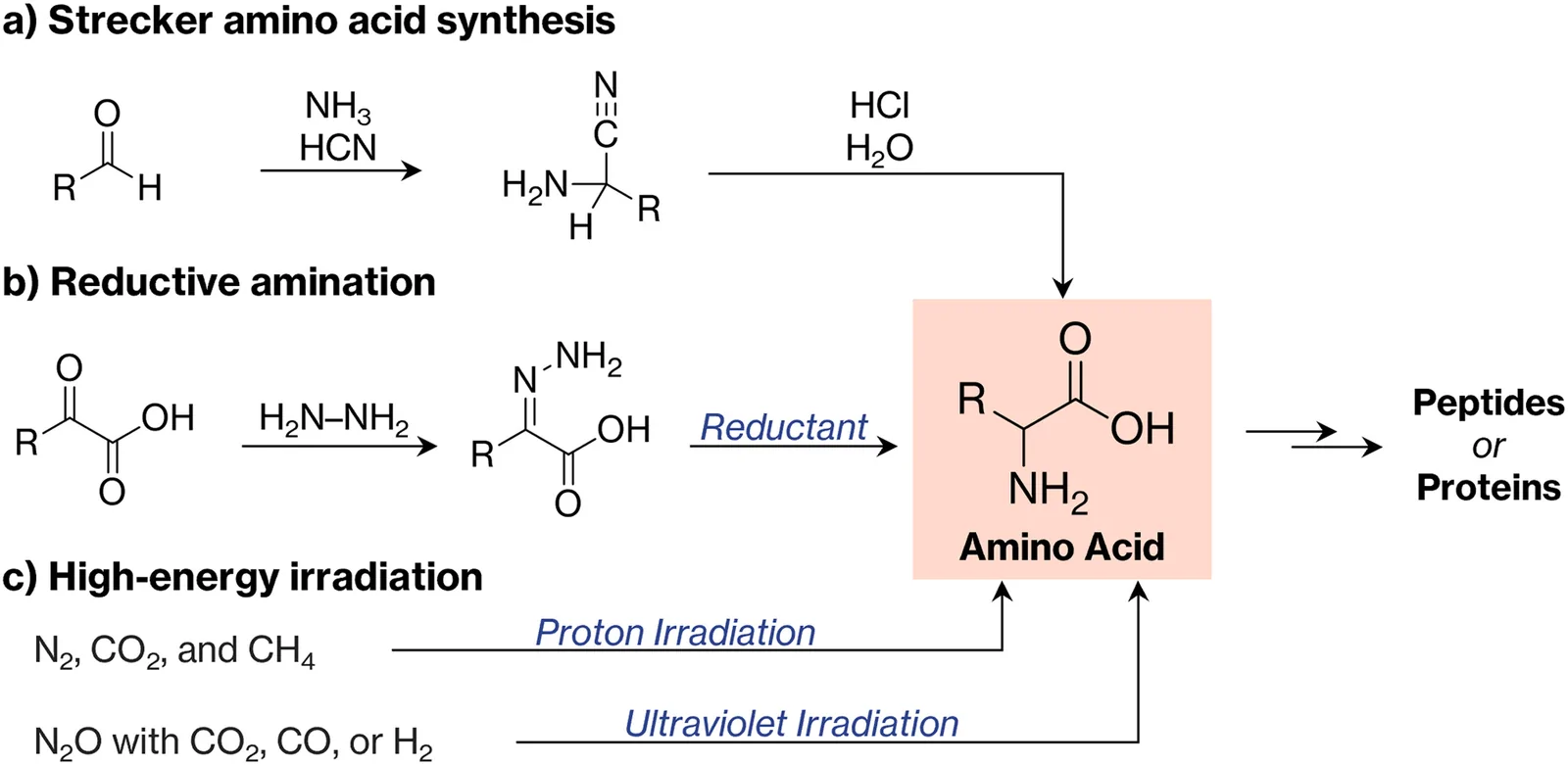
**Some plausible prebiotic chemical synthesis pathways for amino acids.** a) Brief scheme for Strecker reaction. Aldehydes combine with ammonia and cyanide to yield α-aminonitrile, which can then be hydrolyzed to form amino acids. b) Scheme for a typical reductive amination process. Another method for synthesizing amino acids involves the condensation of α-keto acid and an ammonia source (NH_3_, amine, or, in this case, hydrazine). c) Two applications of irradiation in prebiotic chemical synthesis of peptides in reference [29] related to proton irradiation and reference [30] relying on a gas mixture containing nitrous oxide.

Another mechanism for forming amino acids is reductive amination (Fig. 2b). To generate amino acids, condensation of an amine or ammonia with a keto acid first occurs. Then, the imine intermediate is reduced to the amino acid product. Such a reaction can happen at a relatively low temperature and be promoted by metals [28]. If such conditions are prevalent enough on other planets, this pathway would be significant in a broader range of planetary environments.

Moreover, high-energy processes, such as proton or ultraviolet irradiation (Fig. 2c), might have contributed to the formation of prebiotic amino acids. Under proton irradiation in a weak reducing atmosphere consisting of carbon dioxide, methane, and nitrogen, several amino acids have been detected [29]. The products include glycine, aspartic acid, serine, α-aminobutyric acid, and β-alanine. Zang *et al*. also successfully produced glycine, alanine, and serine from gas mixtures containing nitrous oxide (N_2_O) under ultraviolet irradiation [30]. Kuroda and Kobayashi summarized the formation of the 20 proteinogenic amino acids under proton irradiation of the primordial atmosphere, and the scenario was described as three stages: a high-energy reaction stage, an alkylation stage, and a peptide formation stage [31]. For the detailed mechanism of such a gaseous reaction, Li *et al*. utilized a computational chemistry method to explore the reactivity of prebiotic molecules, including CO, H_2_, H_2_O, and NH_3_, illustrating several synthesis pathways for glycine, serine, and alanine [32]. However, the efficiency and yield of these processes are limited in laboratory simulation [29].

Complementary to these pathways, mineral catalysis adds another layer of complexity to the prebiotic synthesis. Catalysis by mineral surfaces has been suggested to play a dual role in prebiotic chemistry, by concentrating reactants from the environment and providing catalytic sites. Chimiak *et al*. reported a prebiotic synthesis pathway of glycine and dicarboxylic amino acids, which starts with nitrate and glyoxylate, and the presence of mineral ferroan brucite [33]. Similar catalytic processes have been found in iron-hydroxide minerals, montmorillonite, olivine, and pyroxene minerals [[34], [35], [36]]. These findings suggest that the composition of minerals on planetary surfaces may significantly impact the local chemistry and, consequently, the potential for prebiotic evolution.

Overall, these insights into amino acid synthesis pathways suggest that multiple mechanisms may be at work. Each mechanism has its own set of constraints and issues that challenge our understanding of chemical evolution.

### 2.2 Nucleobases and nucleotides

Nucleobases and nucleotides are the components of nucleic acids, such as DNA and RNA, which are essential for storing and transferring genetic information in biological systems. Nucleotides, comprising a nucleobase, a pentose sugar (ribose or deoxyribose), and a phosphate group, play vital roles in other cellular processes like energy carriers (in the form of nucleoside triphosphates) and enzymatic catalysis (as the component of cofactors). The detection of purine and pyrimidine nucleobases in carbonaceous meteorites [[37], [38], [39]] indicates their potential formation through abiotic processes in space environments.

Cyanide polymerization (Fig. 3a) was one of the earliest proposed pathways for the prebiotic synthesis of nucleobases [40]. This progress is initiated by the formation of cyanide anion (CN^−^) under basic conditions (pH > 7). These anions can assemble to form iminoacetonitrile (IAN) [41] or other products in mild conditions when the concentration is high enough. The presence of oxygen or ammonia appears to facilitate these reactions [42], raising questions about how variations in ambient conditions might influence the efficiency of such a reaction. Since Oró synthesized adenine from HCN and NH_3_ in the 1960s [43], a range of molecules, including carboxylic acids, after purine, pyrimidines, pteridines, and amino acids have been synthesized via the polymerization of cyanide [44,45].

**Fig. 3:**
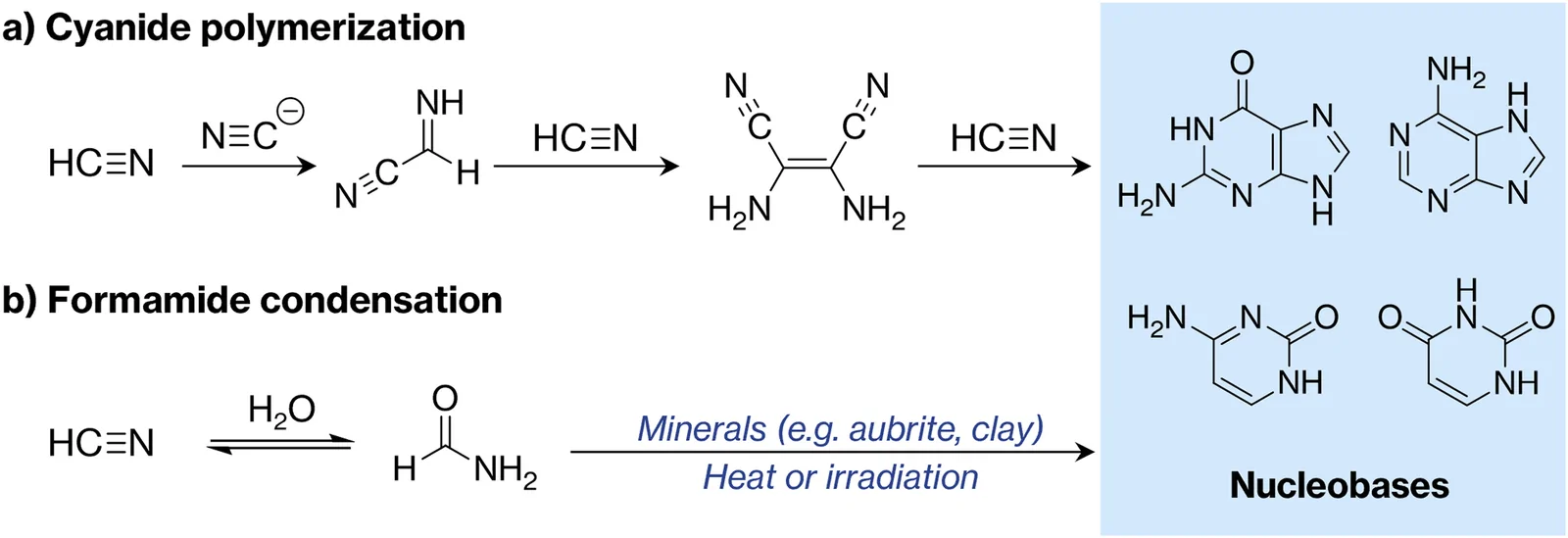
**Prebiotic chemical synthesis pathways for nucleobases starting from cyanide and formamide.** a) Cyanide polymerization, specifically of hydrogen cyanide (HCN). A process where HCN molecules spontaneously link together to form polymers under base-catalysis. The resulting polymers are often complex except for the nucleobases listed. b) Formamide condensation in the prebiotic synthesis of nucleobases. The hydration of HCN yields formamide, whose role in polymerization is more complex. Formamide is involved in the synthesis of nucleic acid bases and the formation of nitrogen-rich polymers when reacted with certain compounds.

Apparently, the availability of HCN is crucial for this synthetic pathway. Several theoretical models for the origin of prebiotic HCN have been proposed recently [46,47], including atmospheric photochemistry, synthesis driven by high-energy impact events, and direct extraterrestrial delivery. Ferus *et al*. utilized a discharge treatment of a reducing atmosphere containing NH_3_, CO, and H_2_O, which could yield a significant amount of formamide and HCN [46]. Subsequent theoretical calculations further corroborate the possibility of this pathway for HCN sources driven by impact plasma or electric discharge [46]. Besides, Zahnle *et al*. studied some photochemical evolution of impact-generated reduced atmospheres, and the production of HCN could occur through nitrogen photolysis [47]. Cyanide or its products have also been detected on asteroids [4,5], comets [6] and meteorites [39], which provides evidence for the direct extraterrestrial delivery of cyanide during the prebiotic period.

The condensation of formamide (NH_2_CHO, the hydration product of cyanide) is also a possible mechanism for the probiotic synthesis of nucleobases or their precursors (Fig. 3b) [48]. The thermal dehydration of formamide can produce reactive intermediates such as formamidine, especially in the presence of catalysts such as minerals, including montmorillonite and aubrite [49]. Recent research employing computational simulations of chemistry in impact craters in conjunction suggests that the meteor impacts and subsequent post-impact formamide thermochemistry can support a continuous prebiotic synthesis of nucleobases [50].

### 2.3 Lipids

Lipids occupy a central role in cellular structure, owing to their unique ability to self-assemble into membranes. They can also serve as long-term energy storage and signal transducers (e.g., steroids) for living organisms [51]. Cellular membranes commonly consist of amphiphilic lipids named glycerophospholipids. These amphiphilic molecules contain a polar head, typically a phosphate group, and two nonpolar “tails” of hydrocarbons. Some amphiphilic lipids spontaneously arrange into vesicles. This property of lipids is essential for the chambering of chemical reaction areas, enabling more efficient biochemical processes by increasing the local concentration of reactants, and protecting the internal components of cells [52]. Understanding the prebiotic synthesis of lipids may provide insights into how primitive membranes formed in the prebiotic environment. In addition, it offers new perspectives on interpreting components of extraterrestrial biosignatures [53].

Fatty acids and glycerol are fundamental components of lipids. Fischer–Tropsch-type (FTT) reactions (Fig. 4a) have been proposed as one of the prebiotic synthesis pathways of fatty acids [54,55]. The Fischer–Tropsch reaction can yield a variety of hydrocarbons from a gaseous mixture of H_2_ and CO. In the prebiotic world, serpentinization can generate serpentine, magnetite, and hydrogen and create an ideal environment, and the key reactant H_2_ for natural FTT reactions [55,56]. This H_2_ can react with carbon monoxide or carbon dioxide to form methane or more complex hydrocarbons. Helge *et al*. assessed the FTT reaction under hydrothermal conditions and confirmed the synthesis routine of linear hydrocarbons, including alkanes, *n*-alkanols, and *n*-alkanoic acids [55]. The FTT reaction can happen in multiple planetary environments, such as hydrothermal vent systems driven by serpentinization [57]. Such mechanisms have been proposed as the plausible causes of the formation of long-chain alkanes recently detected in a Martian sample [58]. However, this process cannot provide a pathway for the synthesis of cyclic or aromatic lipids, such as steroids. This suggests that alternative mechanisms may have contributed to the full complement of lipids in modern life.

**Fig. 4:**
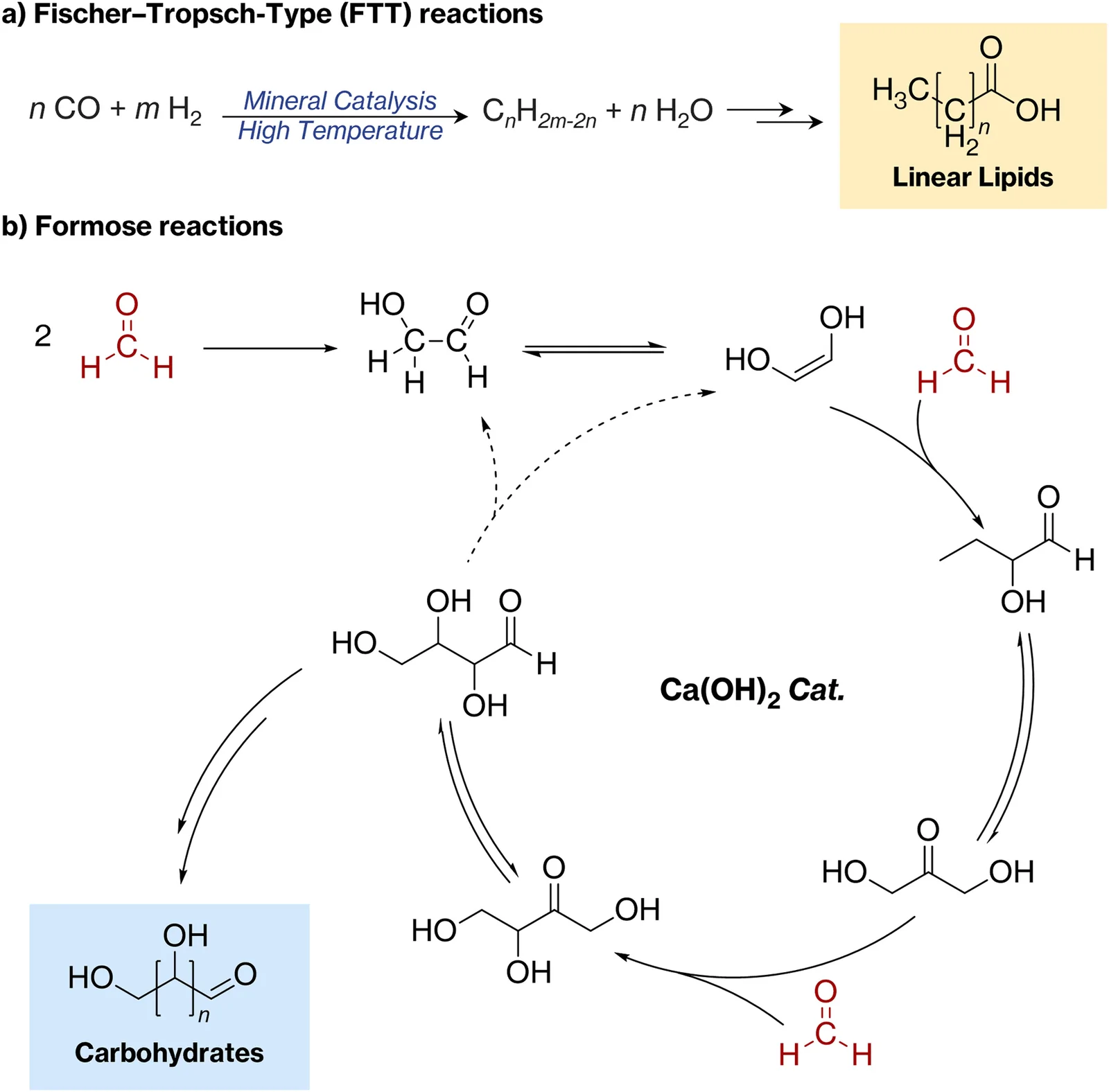
**Fischer–Tropsch-Type (FTT) reactions and Formose reaction.** a) The Fischer–Tropsch synthesis converts a mixture of carbon monoxide and hydrogen into liquid hydrocarbons. Changing specific conditions and catalysts used can result in the formation of fatty acids (linear lipids) and various molecules, including amino acids and hydroxy acids. b) Formose reaction in the synthesis of carbohydrates [70]. The formose reaction involves a set of autocatalytic condensations of formaldehyde (HCHO) to form carbohydrates (sugars).

In parallel, the synthesis of glycerol or its phosphate derivatives is fundamental to the construction of glycerophospholipids. Formamide and formaldehyde have been proposed as plausible precursors of glycerol [59]. The phosphorylation of glycerol is critical, as most of the membrane-forming amphiphile lipids are esters or ethers of glycerol-1-phosphate. Prebiotic pathways for phosphorylation under various conditions have been reported [[60], [61], [62]], including the presence of diamidophosphate (DAP) [60]. The formation of glycerophospholipids involves the attachment of fatty acids to glycerol phosphate, forming phosphatidic acid, which can further evolve into various phospholipids [63]. The diversity of lipids in living organisms also means that no single process can currently fully explain the prebiotic synthesis of lipids.

### 2.4 Carbohydrates

Carbohydrates, especially sugars, serve multiple critical functions in biological systems. For example, glucose provides energy and serves as a structural component for cells, while ribose participates in the formation of nucleic acids. The discovery of diverse sugars and their derivatives in meteorites (especially in hydrated carbonaceous chondrite meteorites) suggests possible extraterrestrial synthesis routes like γ-ray-induced synthesis [64,65]. Yet, skepticism remains whether such high-energy processes could generate and preserve these molecules under the harsh conditions of space [66,67].

The formose reaction (Fig. 4b) is believed to be one plausible mechanism for the prebiotic synthesis of sugars [68,69]. In this process, formaldehyde (HCHO) undergoes alkaline polymerization in the presence of divalent cation catalysts, such as Mg^2+^ or Ca^2+^ [70]. The products of the formose reaction include sugar, sugar alcohols, sugar acids, oligosaccharides, and hydroxy acids [71]. The rate-determined step of this reaction is the generation of glycolaldehyde from the condensation of the first two formaldehydes [69]. However, the formose reaction also results in the degradation or oxidation of the sugars, questioning its relevance to prebiotic synthesis [68,72]. Recent research indicates alkaline submarine hydrothermal vents or minerals may be a plausible location to stabilize formose reaction products [72,73], which responds to the above questioning.

While no single pathway has been conclusively proven to have occurred on early Earth or any other extraterrestrial environments, these models provide a valuable framework for understanding how the building blocks of life might have emerged from simple chemical precursors.

## 3 From molecular detection to understanding emergence of life

The detection of molecular biosignatures on extraterrestrial bodies marks only an initial step toward determining whether life exists, existed, or will exist there. Many organic compounds, considered biosignatures, can form through entirely abiotic processes as stated in Section 2. Apparently, the mere presence of organic molecules is not a definitive signal of life. Instead, what demands examination is the series of chemical processes that could transform such building block molecules into complex biological systems. Understanding these processes in planetary environments is vital, as it not only informs our perspective on the origin of life on Earth but also helps in interpreting information about biosignatures from other extraterrestrial samples.

### 3.1 Formation of complex biological macromolecules

One of the most widely accepted hypotheses for the origin of life on Earth is the theory of chemical evolution [10], which describes the transformation of abiotic molecules into biotic material under specific conditions. The polymerization from simple building blocks or organic monomers to complex biological macromolecules represents a critical step in the emergence of life. These macromolecules include proteins (or polypeptides), nucleic acids, polysaccharides, and their analogs.

The synthesis of protein and peptide polymers is a particularly challenging process under prebiotic conditions. Contemporary biological systems utilize enzyme-catalyzed condensation reactions within ribosomes, where multiple RNAs and precisely arranged ribozymes generate peptide bonds with high fidelity. Although chemical synthesis of protein has been developed for decades, and techniques like native chemical ligation [74] can produce functional proteins easily; however, these methods typically rely on reagents and conditions unlikely to be found in a prebiotic planetary environment.

The formation of peptides requires dehydration condensation, which is thermodynamically unfavorable in an aqueous solution (Fig. 5a). Studies in simulated early Earth scenarios or interstellar environments have demonstrated various pathways for polymerizing amino acids to form peptide chains without the need for biological catalysts. These include mechanochemical dehydration reactions on mineral surfaces, wet-dry cycle models, reaction within microdroplets, and volcanic activity [75,76]. Analogues of amino acids have also been considered as the reactants for peptide synthesis to overcome the activation energy barriers, which have been reviewed in detail [77,78]. The formation of prebiotic peptides might provide primitive catalytic activities that potentially accelerate other prebiotic reactions [79].

**Fig. 5:**
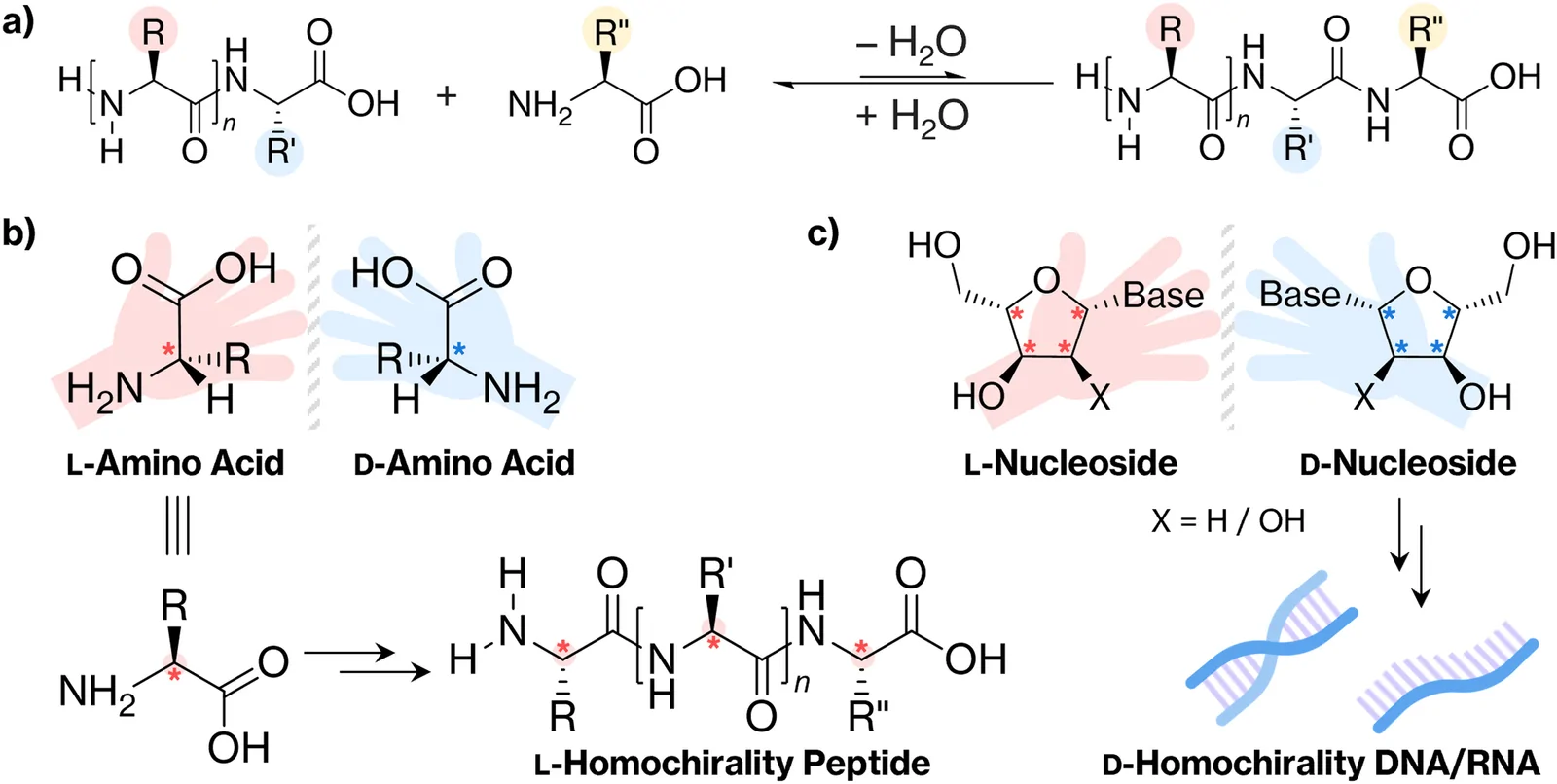
**Formation of biological macromolecules and homochirality from building blocks of life.** a) The polymerization of life’s molecules is extremely unfavorable in aqueous solution. The formation of a peptide bond from amino acids requires dehydration. In contrast, the hydrolysis of its inverse process is thermodynamically favorable in water, which is considered essential to life. b) Chirality is a property of specific molecules, such as a tetrahedral carbon atom bonding with 4 different groups. The l-/d- system is commonly used in biology to denote specific chiral configurations of such molecules. Except for glycine, protein-forming amino acids are chiral, and amino acids produced prebiotically are often racemic mixtures. Peptides or proteins in current life forms are generally homochiral, which means all the amino acid residues are of the l-configurations. b) Nucleosides are also chiral. However, this property is located in the ribose or deoxyribose. Nearly all current living organisms utilize d-nucleosides to form nucleotides and assemble RNA or DNA. * indicates the chiral center.

Nucleic acids (RNA or DNA) are another kind of core biomacromolecule in current life, as described in section 2.2. As for amino acids, the polymerization of nucleotides in aqueous solution is also thermodynamically unfavorable. The biological synthesis of nucleic acids requires templates, enzymes, and a series of cofactors. The reactants for condensation reactions are always nucleotide triphosphates (NTPs). Such synthetic processes would be impossible under prebiotic scenarios. Nevertheless, researchers have revealed plausible routes to template-free polymerization under prebiotic conditions [78,80]. It is also possible that alternative nucleic acid systems with simpler structures than RNA or DNA served as their evolutionary precursors, including peptide nucleic acids (PNAs)and threose nucleic acids (TNAs), whose simpler synthetic pathways might have reduced the activation energy barriers [81,82]. It is worth questioning, however, whether these alternative molecules are capable of efficient information transfer and self-replication, which are required for the evolution of life.

### 3.2 Chirality in biomolecules

Chirality is a fundamental geometrical property of specific molecules, in which the molecules and their mirror images cannot superimpose on each other. In the d/l notation system of stereochemistry, chiral molecules can be either “right-handed” (d-configuration) or “left-handed” (l-configuration), which are mirror images but cannot be perfectly superimposed. Many organic molecules (such as amino acids and sugars) possess a tetrahedral carbon bonded to four different substituents, making that central carbon atom chiral. Furthermore, a system composed of a single enantiomer is described as homochiral, whereas a system containing a 1:1 mixture of both enantiomers is termed racemic.

The predominance of homochirality in biomolecules is a distinctive feature of current life. In nearly all existing life forms, l-amino acids (Fig. 5b) form proteins and d-nucleotides (d-ribose, to be more precise, Fig. 5c) for nucleic acids. If the monomers of biological macromolecules were truly racemic, the formed polymers would be problematic because mixtures of enantiomers would tend to disrupt the specific folding and base-pairing interactions essential for function [83]. Although their enantiomeric isomers (l-sugar or d-amino acids) have been discovered in various functions in prokaryotes, they commonly have specific functions. The transition from a racemic chemical world to homochiral biochemistry raises the question of whether homochirality emerges during the assembly and evolution of biological macromolecules.

Multiple models have been proposed for the development of homochirality in prebiotic contents. One influential proposal focuses on asymmetric autocatalytic polymerization [84,85], a process where a polymer preferentially incorporates monomers that match its intrinsic chirality. This model commonly assumes that the monomers would exist in an inherent enantiomeric excess, even if the difference in abundance of the two isomers is extremely slight. The Soai reaction serves as a powerful experimental demonstration of this principle [86]. Besides, l-enantiomeric excess of isovaline, a non-proteinogenic amino acid, has been observed in carbonaceous chondrites [87], and the asymmetric autocatalytic Soai-like reaction has also been used to explain this chiral preference [88]. However, its direct relevance to prebiotic chemistry is limited, as the reaction conditions are not considered geochemically plausible, nor is it a polymerization process [84].

Several mechanisms have been proposed for generating the initial asymmetry. These include circularly polarized light, magnetic fields, and even the precipitation of heterochiral selectivity products that may induce the homochirality selection in aqueous solution [[89], [90], [91]]. Surface-mediated synthesis on mineral surfaces offers an alternative approach to achieving homochirality. In a non-equilibrium state, chiral enantiomers of amino acids are selected by gypsum [92] and hydroxylated quartz [93]. Recently, spin-polarized electrons have also been proposed as a perspective on the origin of life’s chiral selection, suggesting that electron spin could confer configurational selectivity to non-chiral precursors in environments consistent with primordial Earth conditions, enabling the formation of chiral molecules [91].

Though the initial symmetry imbalance is necessary for homochirality enrichment, the more important thing is the mechanism for chiral amplification and maintenance of this disequilibrium [85]. Therefore, investigating the chemical and geological conditions that promote homochirality is as crucial as searching for chiral molecules themselves. Identifying such conditions and treating them as a class of potential biosignatures will deepen our understanding of life’s origins and guide our search for life beyond Earth.

### 3.3 Origins of genetic systems

A characteristic of modern life is its system for transferring and replicating genetic information. This system is governed by the genetic code, a nearly universal set of rules that describes the correspondence between nucleotide triplets (codons) and amino acids during protein biosynthesis [94]. The near-universality of this code across terrestrial life suggests a single common origin, making its foundational principles a potential biosignature in the search for life elsewhere. Despite its ubiquity, the origin of the genetic code has remained a profound puzzle since its decipherment [95]. Consequently, understanding the fundamental factors that shaped its initial formation and subsequent evolution continues to be a central and active area of investigation.

Several major hypotheses attempt to explain this origin. The RNA world hypothesis presents a stage in life’s evolution in which RNA molecules served both catalytic and information storage functions before the emergence of the DNA-RNA-protein system [96]. The stereochemical hypothesis of the genetic code considers that specific amino acids have chemical affinities for their codons or anticodons [97]. In contrast, the coevolution theory suggests the genetic code evolved alongside amino acid biosynthesis pathways and initial associations between specific amino acids and nucleotides based on physicochemical interactions, which later expanded and standardized through selection pressures favoring translation accuracy and efficiency [98,99]. However, for now, no single theory provides a complete, experimentally validated explanation for how the genetic system emerged in a prebiotic environment.

### 3.4 Chemical information co-evolution

The origins of prebiotic synthesis, homochirality, and the genetic code are three cornerstone problems in the study of life’s origins. Yet, conventional research, often constrained by experimental limitations, has typically addressed these issues in isolation. Drawing inspiration from the ribosome-driven translation process in modern cells, our research introduces an integrated framework that unifies these three problems [100]. This approach positions prebiotic synthesis as the central, linking process. We hypothesize that this synthesis was not merely a step in forming material building blocks, but also played a critical role in concurrently driving the selection of information-bearing properties, namely molecular chirality and the foundational rules of codon-amino acid pairing.

We refer to this integrated framework as *Chemical Information Co-evolution* (CICE). The core principle of CICE is that the synthesis of matter and the regulation of information were not sequential or independent events, but rather two facets of a single, intertwined process (Fig. 6). Under this model, the synthesis of matter is investigated by simulating plausible early Earth conditions, such as fluctuating pH, temperature, mineral catalysis, radiation, and wet-dry cycles, to drive the formation of organic compounds. Simultaneously, the regulation of information is observed by analyzing how these very synthetic processes intrinsically favor one chirality over another (chiral selection) and promote specific, non-random associations between amino acids and nucleotides (the origin of the genetic code). Thus, CICE posits that prebiotic chemistry, much like an evolving language, was not simply generating a random vocabulary of molecules, but was simultaneously writing the grammatical rules that governed their meaningful interactions. By adopting this unifying perspective, we aim to provide a deeper understanding of the intricate processes that governed the chemical evolution of life.

**Fig. 6:**
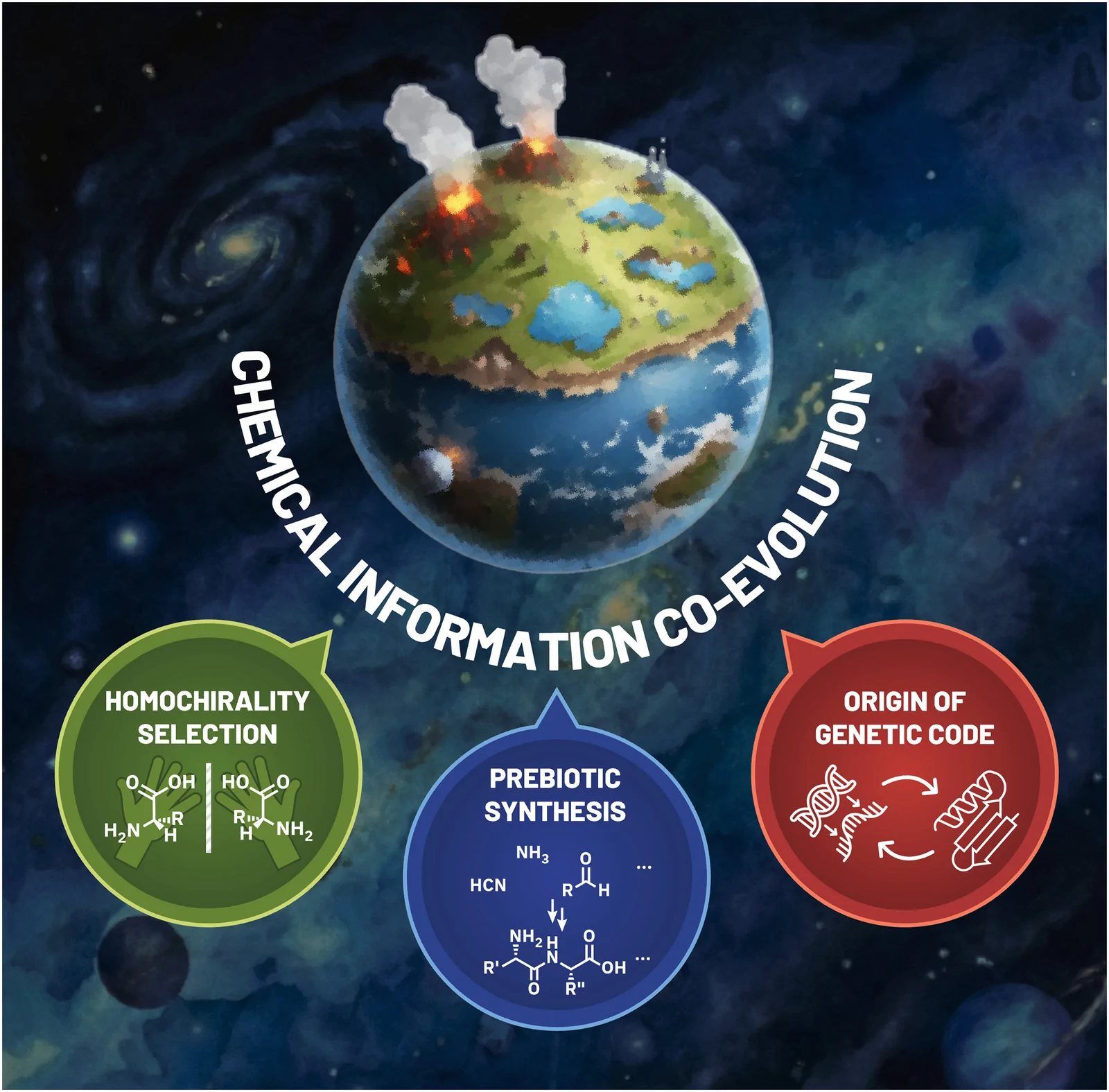
**Diagram of Chemical Information Co-Evolution.** This diagram illustrates the Chemical Information Coevolution (CICE) framework, which treats the prebiotic synthesis and the regulation of information as interrelated key processes in the origin of life. Under simulated early Earth conditions, biomolecules form through prebiotic synthesis (blue). During the synthesis of these substances, homochirality selection (green) may have co-occurred or been regulated by the early genetic code (red). The CICE theory posits that these processes did not occur independently or sequentially, but rather co-evolved, thereby explaining the complex pathway from molecular detection to the emergence of life.

## 4 Implications for extraterrestrial life detection

### 4.1 Refining biosignature interpretation of extraterrestrial life

Chemical, physical, or geological characteristics related to past and present life on Earth are often considered biosignatures of possible extinct or extant extraterrestrial life [101]. While the presence of organic molecules like amino acids is a primary focus, it is increasingly recognized that their mere detection is not a definitive signal of life [9,102], as robust evidence from meteorite analyses and laboratory simulations demonstrates that complex organics can be synthesized through purely abiotic processes [39,103]. But understanding these molecules and their potential prebiotic chemical synthetic pathways will significantly enhance our ability to interpret and expand potential biosignatures. Knowledge of prebiotic reaction networks and the conditions under which these chemical processes may occur enables the evaluation of whether detected organic molecules exist in patterns consistent with biological or abiotic origins. Consequently, the central challenge has shifted from simply finding organic matter to distinguishing a truly biological signal from a product of abiotic processes. To address this ambiguity, the scientific focus has moved beyond searching for a single molecule to identifying suites of evidence and contextual patterns that are collectively difficult to explain without biology. This includes examining relative abundances, structural distributions, homochirality, patterns, and spatial associations with environmental features [[104], [105], [106]].

A prime example is homochirality [104]; while small enantiomeric excesses can be generated abiotically, the near-total preference seen in terrestrial life (e.g., for l- amino acids) could represent a powerful biosignature that is extremely difficult to replicate through known abiotic chemistry alone. Similarly, the discovery of monomers alongside other molecular classes with specific, non-random distributions, such as lipids with a strong preference for even-numbered carbon chains or isoprenoids pattern, would build a much stronger case for a biological origin [107]. Beyond the molecular scale, a key type of biosignature is a significant chemical disequilibrium on a planet. The sustained coexistence of large quantities of reactive gases like oxygen and methane in Earth’s atmosphere, for example, is a robust biosignature implying a massive, continuous biological source that counteracts their natural destruction [108].

Ultimately, understanding the whole landscape of prebiotic reaction networks is not a reason to doubt the search for life, but an essential prerequisite for it. By characterizing the molecules, patterns, and disequilibria that abiotic processes can create, we sharpen our ability to recognize a signal that deviates from this non-living baseline, enabling a sophisticated, probabilistic assessment of its origin [9,109]. Identifying environments where disequilibrium is maintained is thus a dual strategy: it points to locations where the prebiotic synthesis that could lead to life might occur, and it reveals phenomena that could be the product of life itself.

### 4.2 Expanding laboratory simulations of extraterrestrial environments

Laboratory simulations of extraterrestrial environments are an essential component of modern astrobiology. These simulations are designed to recreate conditions thought to be present on other astronomical objects, examining how chemical and physical processes could drive the synthesis of prebiotic molecules. The central target is to understand both the potential for life elsewhere and the pathways leading to the origins of life on early Earth. Many simulation devices are engineered to replicate low atmospheric pressures, extreme temperature ranges, controlled gas mixtures, mineralogical composition, or radiation in space [110,111]. For example, Kobayashi *et al*. utilized proton irradiation to simulate galactic cosmic rays to provide the energy source for amino acid synthesis [29]. Marks *et al*. used an ultra-high vacuum chamber with a helium refrigerator to produce interstellar ice analogs to investigate prebiotic molecules [111]. By carefully modulating these variables, researchers examine how these specific conditions affect chemical reaction pathways.

Considering that mineral surface catalysis plays a central role in several prebiotic chemical pathways, researchers have also utilized mineral composition as a crucial factor in simulating prebiotic environments. A mixture of olivine and pyroxene with salts is used as a simulant of ordinary chondrites to investigate their potential catalysis of amino acid synthesis in Titan’s prebiotic chemistry [36]. Absorption of alanine on olivine or montmorillonite has also been examined experimentally and computationally as a model to explain the persistence of amino acids on meteors [35].

However, while these approaches have been highly successful in demonstrating the formation of life’s fundamental building blocks, a significant gap remains in understanding how these individual components assemble into functional, self-sustaining chemical networks under plausible planetary conditions. The crucial challenge is shifting from cataloguing discrete synthetic pathways to elucidating the principles that govern the transition from a collection of simple molecules to integrated, evolving systems. To bridge this gap, emerging experimental and computational approaches are becoming increasingly vital.

One powerful experimental strategy is the adoption of a systems chemistry perspective. Instead of targeting the synthesis of a single class of molecules, this approach investigates how precursors for multiple essential building blocks can emerge concurrently from a common geochemical scenario [21]. For instance, research by Sutherland and colleagues has demonstrated that simple feedstocks like hydrogen cyanide (HCN), under conditions involving UV radiation and phosphate minerals, can lead to a unified chemical pathway that produces precursors for RNA, proteins, and lipids, suggesting a common origin for these key components of life [20]. This integrated approach provides a more realistic model for how a “protometabolism” might have emerged from a complex environmental chemical mixture.

In parallel with such conceptual shifts, more integrated simulation facilities are being built to simulate multiple environmental parameters together, such as radiation, atmospheric composition, and pressure on Mars or other specific planetary surfaces [112]. Although most simulators were built initially to serve real space missions (e.g., *in situ* detection of planetary compositions [113] or planetary habitability studies [114]), these integrated simulators can also provide specific chemical reaction conditions for the study of prebiotic chemical processes [115].

A key advantage of these facilities is their ability to simulate dynamic and non-equilibrium environments, which are critical for testing the progression from building blocks to polymers and organized structures. Planetary surfaces are not static. Wet-dry cycles, mimicking tidal zones or evaporating ponds on early Earth or Mars, have been shown experimentally to be exceptionally effective at driving the polymerization of amino acids into peptides and nucleotides into RNA-like chains, overcoming the high-energy barrier and unfavorable thermodynamics of condensation reactions in dilute aqueous solutions [116,117]. Similarly, creating sustained geochemical gradients (e.g., pH, temperature, or redox gradients) like those found in hydrothermal vent systems provides a continuous flow of energy to drive chemical reactions far from equilibrium [109,118]. Such non-equilibrium conditions are considered essential for maintaining and evolving complex chemical networks, thereby preventing them from reverting to simple components.

Complementing these advanced experimental setups, computational modeling offers powerful tools to explore a vast chemical space that is inaccessible through laboratory experiments alone. These models can simulate the evolution of chemical species over time, identifying potential autocatalytic cycles [119] or mechanisms for chiral amplification [90]. A second category of models, geochemical models, integrates parameters of planetary environments to help identify whether specific planetary conditions are conducive to the emergence of prebiotic chemistry [120]. By combining kinetic and thermodynamic data from laboratory simulations with environmental parameters from extraterrestrial exploration, these computational approaches can help predict which chemical pathways are most likely to become dominant and self-sustaining on a given planetary body.

In summary, more integrated simulation facilities that combine multiple environmental parameters are essential. Applying these facilities and a systems-level perspective to prebiotic chemistry studies will be paramount to enhancing our understanding of how life’s molecules not only formed, but also organized into the evolving systems that define life itself.

## 5 Conclusion

The search for extraterrestrial life through biosignature detection is a complex scientific endeavor that extends far beyond a simple search for specific molecules. Prebiotic chemistry provides an essential context for interpreting potential biosignatures by illuminating the complex chemical evolution required for the origin of life. Understanding how simple organic precursors may form complex, organized macromolecules and systems under specific environmental conditions is essential for accurately assessing whether a planetary environment has harbored, currently hosts, or could potentially support life.

By combining the search for extraterrestrial life with prebiotic chemistry research, we can develop more reliable methods for interpreting biosignatures and gain a deeper understanding of the fundamental nature of life and its role in the universe’s chemical evolution. Due to the diversity of extraterrestrial conditions, simulating these environmental features accurately in laboratories will contribute to the development of prebiotic chemistry. This will significantly inform the design of future extraterrestrial exploration programs, enabling prebiotic chemistry and the search for extraterrestrial life to be mutually reinforcing. This interdisciplinary approach may ultimately lead us to answer one of humanity’s most profound questions: Are we alone in the universe?

## CRediT authorship contribution statement

**Fude Chen:** Writing – original draft, Visualization, Investigation. **Yufen Zhao:** Supervision, Funding acquisition, Resources. **Jianxi Ying:** Writing – review & editing, Supervision, Funding acquisition, Conceptualization.

## Declaration of competing interest

The authors declare that they have no conflicts of interest in this work.
